# Measurement of 8-hydroxyguanine as an oxidative stress biomarker in saliva by HPLC-ECD

**DOI:** 10.1186/s41021-018-0095-2

**Published:** 2018-04-04

**Authors:** Kazuaki Kawai, Hiroshi Kasai, Yun-Shan Li, Yuya Kawasaki, Shintaro Watanabe, Masanori Ohta, Toru Honda, Hiroshi Yamato

**Affiliations:** 1 Department of Environmental Oncology, Institute of Industrial Ecological Sciences, University of Occupational and Environmental Health, 1-1 Iseigaoka, Yahatanishi-ku, Kitakyushu, Fukuoka, 807-8555 Japan; 2 Department of Health Development, Institute of Industrial Ecological Sciences, University of Occupational and Environmental Health, Japan, 1-1 Iseigaoka, Yahatanishi-ku, Kitakyushu, Fukuoka, 807-8555 Japan; 3 Department of Food and health Sciences, International Collage of Arts and Sciences, Fukuoka Women’s University, 1-1-1 Kasumigaoka, Higashi-ku, Fukuoka, 813-8529 Japan

**Keywords:** Oxidative stress, Biomarker, 8-hydroxyguanine, Saliva, HPLC-ECD

## Abstract

**Introduction:**

Oxidative stress leads to many kinds of diseases. Currently, urinary 8-hydroxydeoxyguanosine (8-OHdG) is widely measured as an oxidative stress biomarker. There is a specific advantage if saliva can be used as the sample to measure the oxidative stress biomarker, because saliva is much easier to collect than urine. In this study, we investigated the measurement of 8-hydroxyguanine (8-OHGua) as an oxidative stress marker in saliva, by a column switching HPLC system equipped with an electrochemical detector (HPLC-ECD).

**Findings:**

The 8-OHGua in saliva could be detected as a single peak by HPLC-ECD. The average level of 8-OHGua in saliva was 3.80 ng/mL in ordinary, non-smoking subjects. The salivary 8-OHGua levels of smokers were significantly higher than those of non-smokers.

**Conclusions:**

Salivary 8-OHGua may be a useful noninvasive and promising oxidative stress biomarker.

## Introduction

Oxidative stress is well known to be a possible cause of many diseases, such as cancer. It is quite useful to know the oxidative status in the body, for the prevention of diseases and aging brought on by oxidative stress. To assess the oxidative status in the body, several biomarkers for oxidative stress have been measured. Among them, urinary 8-hydroxydeoxyguanosine (8-OHdG) is currently the most popular biomarker [1, 2]. 8-Hydroxyguanine (8-OHGua, the base moiety of 8-OHdG) is produced as a result of (I) the oxidation of the guanine base by reactive oxygen species (ROS) within a living organism, (II) the repair of oxidized DNA by glycosylase, or (III) the hydrolysis of a glycosidic bond in oxidized DNA, RNA and the nucleotide pool [1, 3]. The 8-OHdG in DNA leads to the GC to TA transversion mutation [4]. As a defense mechanism against mutation, the 8-OHdG generated in vivo in the DNA or nucleotide pool is excreted into urine by DNA repair or nucleotide pool sanitization systems [5]. For oxidative stress measurement, saliva is now gaining attention as an effective, noninvasive sample source because it is easy to collect. There are several reports of 8-OHdG levels in saliva [6]. However, the range of 8-OHdG levels in saliva differed, depending on the analytical techniques. The accurate measurement of the 8-OHdG level is quite difficult, due to the low concentration of 8-OHdG in saliva. In most of the reports, the salivary 8-OHdG levels were analyzed by ELISA. The values obtained by the ELISA method seemed to be higher than those obtained by the other method. One disadvantage of the ELISA method is that it has a continual problem with the cross-reactivity of the antibody [7]. We have previously reported urinary 8-OHGua as an oxidative stress marker candidate [8]. The diabetic model mouse strain has higher levels of urinary 8-OHGua [9]. In addition, the 8-OHGua levels in serum were increased with X-ray irradiation of mice [10]. In general, the concentration of 8-OHGua in biological samples is higher than that of 8-OHdG. In this study, we established an analytical method with an HPLC-ECD system to measure the salivary 8-OHGua level, as an oxidative stress marker.

## Methods

Collection of saliva: About 5 mL of passive drool from normal healthy human subjects were collected in the morning (9 A.M. – 11 A.M.), after rinsing the mouth with water. The saliva samples from smokers (6 males, ages: 45–55, duration of smoking: 10–20 years, number of cigarettes smoked: 10–20/day) and the controls (non-smokers, 3 males and 3 females, ages: 35–65) were directly collected into polypropylene tubes. The saliva was stored at − 20 °C until use.

Analysis of 8-OHGua in saliva: The saliva (0.6 mL) was digested with proteinase K (30 μL of 20 mg/mL solution, Wako Chemical, Tokyo, Japan) at 37 °C for 1 h. The proteinase-digested saliva was evaporated to dryness with a vacuum centrifuge, and dissolved in 300 μL of a diluent (1.8% acetonitrile, 62 mM NaOAc (pH 4.5) and 0.01 mM H_2_SO_4_). After ultrafiltration with a centrifugal filter (Amicon Ultra, Ultracel-10 K, Merck Millipore Ltd., Darmstadt, Germany), a 20 μL aliquot of the filtrate was injected to the HPLC system.

The 8-OHGua level in saliva was determined using the column switching HPLC-ECD system. The HPLC conditions and apparatus are basically similar to the automated method for the urinary 8-OHdG analysis [11]. The HPLC system consists of three pumps, the sampling injector, two valves, the HPLC-1 column, the HPLC-2 column, and the EC detector. The HPLC-1 column was set in a column oven at 65 °C, and the HPLC-2 column was set at 25 °C. A 20 μL aliquot of the saliva sample was injected into HPLC-1 (MCI GEL CA08F, 7 μm, 1.5 × 30 mm (guard column) + 1.5 × 90 mm, solvent A, 60 μL/min) by the sampling injector. In this method, the 8-OHGua fraction was collected depending upon its retention time, and was automatically injected into the HPLC-2 column (GL Sciences, InertSustain C18, 3 μm, 4.6 × 250 mm, solvent B, 0.6 mL/min). This column was coupled with an ECD (ECD-300, Eicom Co., Kyoto, Japan, applied voltage: 550 mV). The solvents used were: solvent A, 2% acetonitrile in 0.3 mM sulfuric acid; solvent B, 9 mM K_2_HPO_4_, 25 mM KH_2_PO_4_, 0.5 mM EDTA•2Na, 2.5% acetonitrile. After each saliva injection, the guard column was reverse cleaned automatically with 0.5 M ammonium sulfate: acetonitrile (7:3, *v*/v). The amount of protein in each saliva sample was determined with the Bradford reagent (Bio-Rad protein assay).

Analysis of 8-OHdG in saliva: For the analysis of 8-OHdG in saliva, 1 mL of saliva was pretreated with an 8-OHdG pretreatment kit (TANITA, INC., Tokyo, Japan) according to the product manual. The pretreated sample was concentrated 5-fold with a vacuum centrifuge. The concentrated sample was injected into an HPLC column (Capcell Pak C18 MG, 3 μm, 4.6 × 250 mm, Shiseido Fine Chemicals, Tokyo, Japan) equipped with UV (UV-8020, Tosoh Co., Tokyo, Japan) and ECD (ECD-300, Eicom Co., Kyoto, Japan, applied voltage: 550 mV) detectors. The mobile phase was 10 mM NaH_2_PO_4_, containing 8% methanol and 0.13 mM Na_2_EDTA, delivered at a flow rate of 0.7 mL/min. The column temperature was 32 °C.

The study was approved by the Ethics Committee of Medical Research, University of Occupational and Environmental Health, Japan.

### Statistical analysis

Analyses were performed using the Pharmaco Basic software (Scientist Press Co., Tokyo, Japan).

## Results

**Fig. 1:**
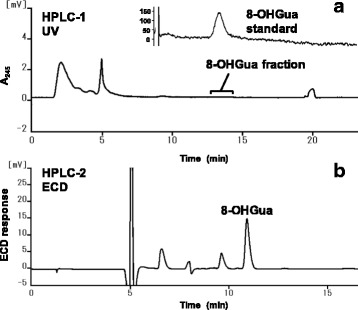
**a** HPLC-1 chromatograms of the 8-OHGua standard and a human saliva sample. **b** HPLC-2 chromatogram of the 8-OHGua fraction of human saliva obtained by HPLC-1

**Fig. 2:**
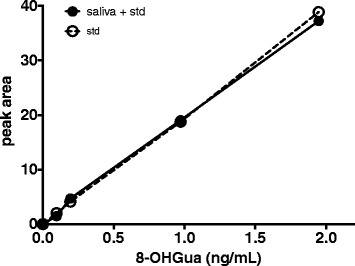
Standard curve of 8-OHGua obtained from the 8-OHGua standard in the diluent (1.8% acetonitrile, 62 mM NaOAc (pH 4.5) and 0.01 mM H_2_SO_4_) (open circles) and curve of the 8-OHGua-spiked saliva (closed circles; the original 8-OHGua peak level of an authentic saliva sample (20.64) was subtracted)

**Fig. 3:**
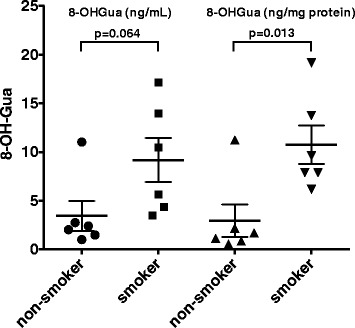
Salivary 8-OHGua levels of non-smokers and smokers. Groups were compared with the unpaired Student t test

As seen in the typical chromatogram of 8-OHGua measurement in saliva, the HPLC peak of 8-OHGua was clearly separated as a single peak by the HPLC-ECD system (Fig. 1). The quantification limit was estimated to be below 0.2 ng/mL of saliva, and the recovery percentage was 96.2 ± 8.6 (mean ± S.E.), based on the calibration curve (Fig. 2) in the relatively lower range. The average level of 8-OHGua in saliva was 3.80 (1.02–11.04, *n* = 6) ng/mL in the ordinary subjects (non-smokers). The salivary 8-OHGua levels of smokers were higher than those of non-smokers (Fig. 3). There was a statistically significant difference between smokers and non-smokers when the applied unit was defined as ng 8-OHGua per mg protein (Fig. 3). In addition, the average 8-OHdG levels in saliva were 3.3 (1.3–6.7, *n* = 6) pg/mL in the same non-smoking subjects.

## Discussion

Considering its safe and noninvasive collection, saliva is an ideal specimen as compared to blood for most biomarkers. According to data measured by ELISA, the salivary 8-OHdG levels in ordinary subjects were reported to be between 0.68–1.56 ng/mL. In contrast, the levels were 0.010 ± 0.007 ng/mL by an LC-MS/MS analysis [12]. This is a several hundred-fold difference. In this study, the HPLC-ECD analysis results revealed that the average 8-OHdG levels in saliva were quite similar to those obtained by the LC-MS/MS analysis. From our previous report [7], the discrepancy between the ELISA and the chromatographical analysis is likely to be due to the cross-reactivity of the antibody used in the ELISA. It seems that chromatographic techniques are more suitable for 8-OHdG measurements in biological fluids. As the 8-OHdG level in saliva is very low (several pg/mL), the pretreatment of saliva for 8-OHdG measurement is complex and difficult for the accurate measurement of large quantities of clinical samples. In contrast, the 8-OHGua contents in saliva were several hundred-fold higher than the 8-OHdG contents. The levels of salivary 8-OHGua were accurately analyzed by the HPLC-ECD system for all subjects in this study. For the evaluation of a biomarker in saliva, it is unknown whether concentration correction is required. Therefore, the 8-OHGua levels were normalized by the protein levels; however, the effect seemed limited. In this regard, further studies will be needed. To the best of our knowledge, only a few reports of 8-OHGua in saliva have been published. At this time, there is no way to measure the 8-OHGua by ELISA, because an adequate antibody for 8-OHGua is not available. In a previous report [13], the measurement was performed with a relatively expensive LC-MS/MS system. In this study, we have reported the accurate measurement of 8-OHGua levels in saliva by a universal HPLC-ECD system. There is a significant economic benefit for an epidemiological study dealing with a large number of samples. The 8-OHGua in saliva could be a useful noninvasive and promising oxidative stress biomarker, in addition to the urinary 8-OHdG.

## Abbreviations

8-OHdG: 8-hydroxydeoxyguanosine
8-OHGua: 8-hydroxyguanine
ECD: electrochemical detector
